# Analysis of *CDK12* alterations in a pan‐cancer database

**DOI:** 10.1002/cam4.4483

**Published:** 2021-12-12

**Authors:** Elizabeth Pan, Angelo Cabal, Juan Javier‐DesLoges, Devin Patel, Justine Panian, Suzanna Lee, Justin Shaya, Taylor Nonato, Xiaojun Xu, Tyler Stewart, Brent Rose, Ahmed Shabaik, Ezra Cohen, Razelle Kurzrock, Pablo Tamayo, Rana R. McKay

**Affiliations:** ^1^ Division of Hematology‐Oncology Department of Medicine University of California San Diego San Diego California USA; ^2^ Department of Urology University of California San Diego San Diego California USA; ^3^ Moores Cancer Center University of California San Diego San Diego California USA; ^4^ Department of Radiation and Applied Sciences University of California San Diego San Diego California USA; ^5^ Department of Pathology University of California San Diego San Diego California USA; ^6^ Division of Medical Genetics School of Medicine University of California San Diego California USA

**Keywords:** biomarkers, cancer genetics, clinical cancer research, genomics

## Abstract

**Background:**

*CDK12* inactivation leading to increased neoantigen burden has been hypothesized to sensitize tumors to immune checkpoint inhibition. Pan‐cancer data regarding the frequency of *CDK12* alterations are limited. We aimed to characterize *CDK12* alterations across all cancer types through real‐world clinical‐grade sequencing.

**Methods:**

This was a single‐center retrospective analysis of 4994 cancer patients who underwent tissue or blood genomic profiling, including *CDK12* assessment, conducted as part of routine care from December 2012 to January 2020. Prevalence, clinical characteristics, and treatment outcomes of patients with tumors with pathogenic *CDK12* alterations were described.

**Results:**

In all, 39 (0.78%, *n* = 39/4994) patients had pathogenic *CDK12* alterations. Among *CDK12*‐altered tumors, the most common organ site was prostate (*n* = 9, 23.1%) followed by colorectal (*n* = 5, 12.8%). Adenocarcinoma was the most common histology (*n* = 26, 66.7%). Median follow‐up from time of diagnosis was 4.02 years. Median overall survival from time of metastasis was 4.43 years (95% CI: 3.11–5.74). Ten patients with *CDK12*‐altered tumors received at least one immune checkpoint inhibitor‐containing regimen. The majority of patients (*n* = 6/10, 60%) experienced an objective response. Progression‐free survival for patients who had metastatic disease and received a checkpoint inhibitor‐containing regimen was 1.16 years (95% CI: 0.32–2.00).

**Conclusion:**

*CDK12* alterations are rare events across hematologic and solid tumor malignancies. They represent a clinically distinct molecular cancer subtype which may have increased responsiveness to checkpoint inhibition. Prospective studies are warranted to investigate checkpoint inhibition in *CDK12*‐altered tumors.

## Graphical abstract

CDK12 inactivation leading to increased neoantigen burden has been hypothesized to sensitive tumors to immune checkpoint inhibition. Pan‐cancer data regarding the frequency of CDK12 alterations is limited. CDK12 altered tumors represent a clinically distinct molecular cancer subtype which may have increased responsiveness to checkpoint inhibition.

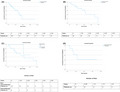

## 1 INTRODUCTION

The growing routine use of molecular profiling in recent years has revolutionized the medical management of advanced hematologic and solid tumor malignancies. One goal of molecular profiling is to identify molecular susceptibilities in different tumors that may be prognostic or predictive of response to treatment, the latter resulting in the practice of precision oncology. Understanding key characteristics of molecular targets present in a variety of tumor types is central to the goal of tailoring effective treatments in the age of precision medicine.

*CDK12* alterations have generated recent interest as a potential biomarker for cancer response. *CDK12* encodes for the tumor‐suppressor protein cyclin‐dependent kinase‐12, which plays various roles in RNA processing and DNA repair in select genes.^1^, ^2^, ^3^, ^4^ *CDK12* has been implicated in the homologous recombination repair pathway, although efforts to target these tumors with poly [ADP‐ribose] polymerase [PARP] inhibitors such as olaparib have achieved mixed results.^5^, ^6^ More recently, *CDK12* loss of function alterations was found to be associated with increased focal tandem duplications and greater genome‐wide structural variation in ovarian and prostate cancers.^7^, ^8^, ^9^ Growing evidence has shown that structural variations resulting from *CDK12* inactivation may result in increased neoantigen burden and increased expression of chemokines, making patients with *CDK12*‐altered tumors promising candidates for immune checkpoint inhibitors.^10^

Given its potential as a target for an expanding panel of therapies, further uncovering the clinical and genomic features of the *CDK12* alteration genotype in the oncologic landscape is growing in importance. In prostate cancer, *CDK12* inactivation has shown more aggressive clinical features including greater proportion of distant metastases and shorter time to PSA progression.^6^, ^11^, ^12^ However, there are limited pan‐cancer data regarding the frequency of *CDK12* alterations, especially in the context of patients with or without metastases. We aimed to characterize the clinical features of *CDK12*‐altered tumors utilizing a pan‐cancer database of patients undergoing clinical‐grade genomic profiling.

## 2 METHODS

### 2.1 Patient population

This was a single‐center retrospective analysis approved by the Institutional Review Board at the University of California San Diego. We evaluated patients with a diagnosis of cancer who underwent clinical‐grade (i.e., Clinical Laboratory Improvement Amendments‐certified) genomic profiling as part of routine care. Eligible patients were patients older than 18 years of age with a diagnosis of invasive cancer who had at least one clinical‐grade next generation sequencing test which included analysis of *CDK12* on either tissue or blood from December 2012 to January 2020.

### 2.2 Genomic data

Patients with evidence of a pathogenic *CDK12* alteration affecting at least one allele were included. Pathogenic alterations were defined as those that result in a truncated protein (i.e., frameshift, nonsense, splicing mutations) or genomic rearrangements that involve the *CDK12* locus (e.g., homozygous deletions, gene fusions, other translocations). Pathogenic mutations were defined using databases such as COSMIC and published literature. A list of pathogenic mutations and classification of alterations is listed in Table S1. A variety of clinical‐grade sequencing assays was used to determine *CDK12* status in our cohort (tissue‐based assays utilizing either primary or metastasis tissue: FoundationOne, FoundationOne CDx, and Tempus xE/xO/xT; cell‐free DNA‐based assays: Guardant360, FoundationOne Heme, and FoundationOne Liquid CDx). The average depths of coverage for the various next generation sequencing platforms are as follows: 500x for Tempus xT, 250x for Tempux xE, 300x for Tempus xO, and 15000x for Guardant. The median depths of coverage for Foundation sequencing platforms were 500x for Foundation CDx, 500x for Foundation Heme, and deep coverage for Foundation Liquid CDx (Foundation Medicine does not disclose the exact liquid coverage, but it is significantly deeper than tissue due to low levels of circulating tumor DNA in the plasma). Co‐occurring alterations in other genes for patients with *CDK12*‐altered tumors were analyzed. Microsatellite instability (MSI) and mismatch repair (MMR) status was not included in the analysis due to limited data provided by the earlier genomic profiling reports. Analysis was only performed on co‐occurring alterations that were present on the same assay in which a *CDK12* alteration was detected. If patients had more than one genetic test performed, the most recent tissue‐based test was utilized for analysis. Matched samples were not analyzed.

### 2.3 Clinical data

Baseline demographic, pathologic, and clinical characteristics were abstracted from the electronic medical record. Data regarding age, gender, race/ethnicity, smoking history, cancer site of origin, histology, and Charlson Comorbidity Index^13^ were captured. The American Joint Committee on Cancer staging system 8th edition was used to define patients with localized, regional, and distant metastases.^14^ We tabulated systemic treatments with a focus on immune checkpoint inhibitors.

### 2.4 Statistical analysis

Patient and disease characteristics were summarized using descriptive statistics. The prevalence of patients with *CDK12* alterations was calculated. Overall survival (OS) was defined as the date of diagnosis to death, censored at the time of last follow‐up. We also calculated OS from the date of metastasis to death, censored at the time of last follow‐up. Progression‐free survival (PFS) was defined for patients receiving immune checkpoint inhibitors as the time from treatment initiation with first‐line immunotherapy to radiographic progression (using the Response Evaluation Criteria in Solid Tumors [RECIST] version 1.1 principals), clinical progression (defined as disease‐related complication or clinical deterioration) or death, whichever occurred first. Response was assessed using RECIST version 1.1 principals. Time‐to‐event outcomes (PFS, OS) were analyzed using the Kaplan–Meier method, and median values (with 95% confidence intervals [CI]) were reported. As an exploratory analysis, we compared the OS using the log‐rank test in patients with metastatic disease having received a checkpoint inhibitor compared to those with metastatic disease not having received a checkpoint inhibitor. Tumor mutational burden for patients having received a checkpoint inhibitor was stratified as low (≤5 mutations/Mb), intermediate (>5 and <20), high (≥20 and <50), and very high (≥50).

## 3 RESULTS

### 3.1 Patient characteristics

In this analysis of 4994 patients with cancer having undergone clinical‐grade genomic sequencing, 39 (0.78%) were identified to have pathogenic *CDK12* alterations (Table S2). Of patients with metastatic disease (*n* = 2997), 1.1% (*n* = 32) were identified to a pathogenic *CDK12* alteration compared to 0.36% (*n* = 7) in patients with localized disease (*n* = 1937).

Patient and disease characteristics of patients with *CDK12*‐altered tumors are detailed in Tables 1 and 2, respectively. The median age at diagnoses was 64 years (interquartile range: 58–71 years) and the majority of patients were male (*n* = 25, 64.1%). While 61.5% of patients (*n* = 24) were white, 38.5% (*n* = 15) were non‐white with Hispanic (*n* = 6, 15.4%) representing the next most frequent racial group represented in the cohort of patients with *CDK12*‐altered tumors. Most patients (*n* = 18, 46.2%) had localized disease at diagnosis of whom 15 subsequently developed metastatic disease. A total of 33 patients (84.6%) developed metastatic disease at any time during their disease course. The most common histology was adenocarcinoma (*n* = 26, 66.7%). *CDK12* alterations were observed across a spectrum of malignancies, with the most common primary sites being prostate (*n* = 9, 23.1%), followed by colorectal (*n* = 5, 12.8%) and breast (*n* = 4, 10.3%).

**TABLE 1:** Patient characteristics for individuals with *CDK12*‐altered tumors (*n* = 39)

Clinical characteristic	Number or Median	Percent or Interquartile range
Age at diagnosis	64	(58–71)
Gender		
Male	25	64.1%
Female	14	35.9%
Race/Ethnicity		
White	24	61.5%
Asian/Pacific Islander	4	10.3%
Hispanic	6	15.4%
Black	3	7.7%
Multiracial	2	5.1%
American Indian/Alaska Native	0	0.0%
Smoking history		
Never	19	48.7%
Former	19	48.7%
Current	1	2.6%
Charlson Comorbidity Index		
0	0	0%
1	26	66.7%
2	11	28.2%
3+	2	5.1%

**TABLE 2:** Tumor characteristics and treatment exposure for individuals with *CDK12*‐altered tumors (*n* = 39)

Characteristic	Number	Percent
Histology		
Adenocarcinoma	26	66.7
Invasive ductal carcinoma	3	7.7
Hepatocellular carcinoma	2	5.1
Squamous cell carcinoma	0	0.0
Small cell carcinoma	1	2.6
Urothelial carcinoma	1	2.6
Neuroendocrine carcinoma	1	2.6
Melanoma	1	2.6
Lymphoma	1	2.6
Papillary serous ovarian carcinoma	1	2.6
Desmoplastic small round cell tumor	1	2.6
Salivary duct carcinoma	1	2.6
Primary cancer site		
Prostate	9	23.1
Colorectal	5	12.8
Breast	4	10.3
Small Bowel	3	7.7
Lung	1	2.6
Esophageal	3	7.7
Liver	3	7.7
Ovarian	2	5.1
Stomach	2	5.1
Bladder	1	2.6
Gallbladder	1	2.6
Lymphoma	1	2.6
Melanoma	1	2.6
Non‐melanoma skin cancer	1	2.6
Salivary	1	2.6
Uterine	1	2.6
Stage at diagnosis		
Localized	18	46.2
Regional nodal	11	28.2
Metastatic	6	15.4
Unknown	4	10.3
Metastatic disease at any time		
Yes	33	84.6
No	6	15.4
Systemic therapy		
Neoadjuvant/adjuvant treatment for local disease	14	35.9
Systemic treatment for metastatic disease	33	84.6
Cytotoxic chemotherapy	28	71.8
Platinum‐containing chemotherapy	9	23.1
Checkpoint inhibitor‐containing regimen	10	25.6
Tyrosine kinase inhibitor	7	17.9
PARP inhibitor	3	7.7

### 3.2 Genomic characteristics

Among the 39 patients with pathogenic *CDK12* alterations, 33 (84.6%) patients were identified using tissue testing: 30 (90.9%) using FoundationOne and three (9.1%) using Tempus xT. Of patients identified on tissue testing (*n* = 33), 22 (66.7%) were identified from primary tumor testing and 11 (33.3%) from metastasis tumor testing. In the entire cohort of 4994 patients, 0.5% were identified with *CDK12* alterations on primary tumor testing and 0.26% on metastasis testing. Six patients were identified using ctDNA testing: three using Guardant360, one using Tempus xF, one using FoundationOne Heme, and one using FoundationOne CDx Liquid.

**FIGURE 1:**
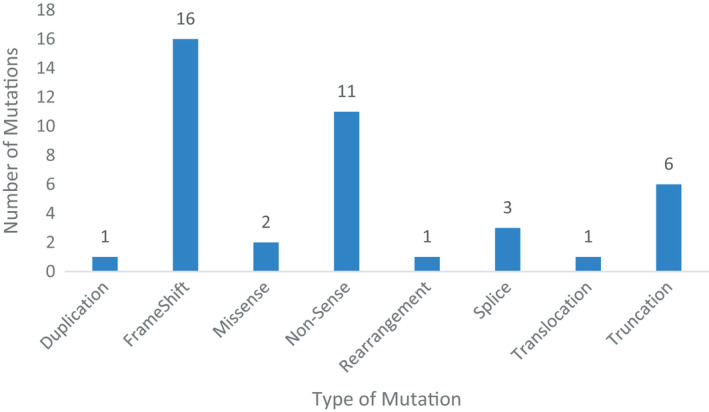
*CKD12* alteration type. There are a total of 41 mutations observed in 39 patients. Two patients had biallelic alterations

**FIGURE 2:**
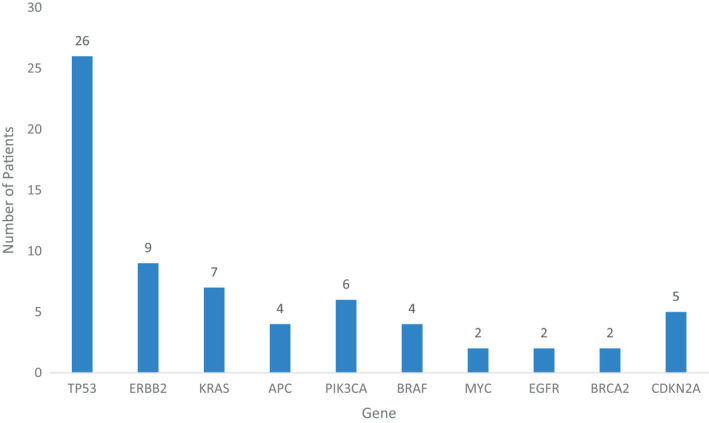
Frequency of genes with co‐occurring alterations in patients with *CDK12* alterations

There were a total of 41 *CDK12* alterations observed across 39 patients, and the alteration types are summarized in Figure 1. Of patients with pathogenic *CDK12*‐altered tumors, two patients (4.5%) had biallelic inactivating alterations. Both patients with biallelic alterations had prostate cancer. The spectrum of the type of *CDK12* alterations observed across primary sites of origin is detailed in Figure 2. The most common types of alterations were frameshift mutations (*n* = 16), followed by non‐sense mutations (*n* = 11), while a smaller proportion of alterations due to other mechanisms. A complete list of all *CDK12* alterations is included in Table S2.

In all, 23 patients had more than one sequencing assay performed throughout their clinical course, and 30.4% (*n* = 7/23) demonstrated concordance with *CDK12* alterations. Of the nine patients who had multiple genetic profiling assays that included at least one prior to treatment initiation, only three patients did not have CDK12 alterations at baseline and acquired it during the course of treatment. We interrogated for the presence of additional concurrent genomic alterations in patients with *CDK12* alterations (Figure 2). Co‐occurring alterations were the most common in *TP53*, occurring in 26 patients (66.6%). This was followed by *ERBB2* in nine patients (23.0%), *KRAS* (*n* = 7/29, 17.9%), and *PIK3CA* (*n* = 6/39, 15.3%). Co‐occurring *BRCA2* alterations were identified in two patients (5.1%), one with gastrointestinal tumors and one with bladder cancer.

Tumor mutational burden (TMB) was evaluated in tumors with *CDK12* alterations. Of the samples with reportable TMB (*n* = 40), the majority had low‐ or intermediate‐range TMB at 45% and 37.5%, respectively. The remaining had high (2.5%) or very high (15%) TMB. The TMB for patients who received immunotherapy are listed in Table 3. Of the patients having received immunotherapy, 81.8% (*n* = 9/11) had at least intermediate TMB, with two patients having high/very high TMB. Notably, patient #9 had a homozygous *CDK12* splice site alteration with co‐occurring CDK6 amplification and EGFR amplification, and had a partial response to pembrolizumab.

**TABLE 3:** Patients with metastatic disease treated with immunotherapy and/or PARP inhibitor and/or platinum compound

Patient	Primary Malignancy	Regimen	Line of therapy for metastatic disease	Best objective response to therapy (CR, PR, SD, PD)	Time to progression or last follow‐up (months)	Develop‐ment of IRAE	TMB^a^	*CDK12* mutation
Checkpoint inhibitor‐based treatment for metastatic disease								
1	Melanoma	Pembrolizumab + dabrafenib + trametinib	2	PR	9	Y	62.3	Q1368: Non‐sense
		Ipilimumab	6	PR	36.2	Y		
2	Gastrointestinal	Pembrolizumab	4	PR	36.5	N	63.2	T1463fs*30+: Frameshift
3	Urothelial carcinoma	Durvalumab + investigational agent	3	PR	41.9	N	18.4	E205: Non‐sense
4	Esophagus	Pembrolizumab + trastuzumab + bevacizumab	4	PD	14	N	6.1	Intron 7 rearrangement: Truncation
		Anti‐PD‐1 monoclonal antibody	5	PD	1	N		
5	Lung	Pembrolizumab	2	PD	7.5	N	13	F336fs*1: Frameshift
		Nivolumab	4	PR	3.7	N		
6	Cutaneous SCC^b^	Pembrolizumab	1	PR	8.9	N	19	Y279: Non‐sense
7	Liver	Nivolumab	1	PR	3.1	N	6	P577fs: Frameshift
8	Prostate	Pembrolizumab	4	PR	12.6	N	8	CDK12 splice site 2610‐20_2610‐1>T
9	Prostate	Nivolumab + ipilimumab + enzalutamide	7	PD	1.9	Y	Unavailable	D416fs: Frameshift
10	Ovary	Pembrolizumab + niraparib	8	PD	3.7	N	6	L760fs*2: Frameshift
PARP Inhibitor‐based treatment for metastatic disease								
2	Gastrointestinal	Olaparib + cisplatin	3	PD	2	NA	63.2	T1463fs*30+: Frameshift
10	Ovary	Pembrolizumab + niraparib	8	PD	3.7	NA	18.4	L760fs*2: Frameshift
12	Colon	Olaparib + trametinib + sulindac + bevacizumab	6	PD	2.8	NA	5	G239: Non‐sense
Platinum‐based treatment for metastatic disease								
2	Gastrointestinal	Olaparib + cisplatin	3	PD	2	NA	63.2	T1463fs*30+: Frameshift
3	Bladder	Cisplatin + gemcitabine	1	PR	2	NA	18.4	E205; Non‐sense
		Carboplatin + gemcitabine	2	PD	4	NA		
10	Ovary	Carboplatin + paclitaxel	1	PR	9.5	NA	6	L760fs*2: Frameshift
12	Colon	Oxaliplatin + capecitabine	1	PR	11.5	NA	5	G239; Non‐sense
13	Breast	Carboplatin + gemcitabine	4	PD	2.5	NA	8	*CDK12* c.2420‐1G>A
14	Ovary	Carboplatin + paclitaxel	2	PR	16	NA	5	Q244s*93; Frameshift
		Carboplatin + gemcitabine	5	PD	2	NA		
15	Lung	Carboplatin + bevacizumab + pemetrexed	1	PR	4.3	NA	13	F336fs*1; Frameshift
16	Gallbladder	Gemcitabine + cisplatin	2	SD	22	NA	4.7	R981; Frameshift
17	Prostate	Carboplatin + cabazitaxel + enxalutamide	6	PD	3	NA	Unavailable	D416fs; Frameshift

Abbreviations: CR, complete response; PR, partial response; SD, stable disease; PD, progressive disease; NA, Not applicable; IRAE, immune‐related adverse event.

^a^ Tumor mutational burden stratified as low (≤5 mutations/Mb), intermediate (>5 and <20), high (≥20 and <50), and very high (≥50).

^b^ Did not have distant metastasis; received immunotherapy for non‐curative, locally advanced unresectable cutaneous SCC.

### 3.3 Treatment exposure

There were 14 (35.9%) patients who received neoadjuvant/adjuvant systemic therapy for local/regional disease and 33 (84.6%) patients who received systemic therapy for metastatic disease. The median number of lines of systemic therapy administered to patients with *CDK12*‐altered tumors was three. Cytotoxic chemotherapy was the most common treatment, with nine patients (23.1%) receiving therapy with a platinum‐containing regimen. Additionally, 17.9% (*n* = 7) and 7.7% (*n* = 3) received treatment with tyrosine‐kinase inhibitors and PARP inhibitors, respectively. A total of 10 patients (25.6%) received treatment with at least one checkpoint inhibitor‐containing regimen, with three patients receiving two checkpoint inhibitor‐containing regimens (Table 2).

### 3.4 Treatment outcomes

**FIGURE 3:**
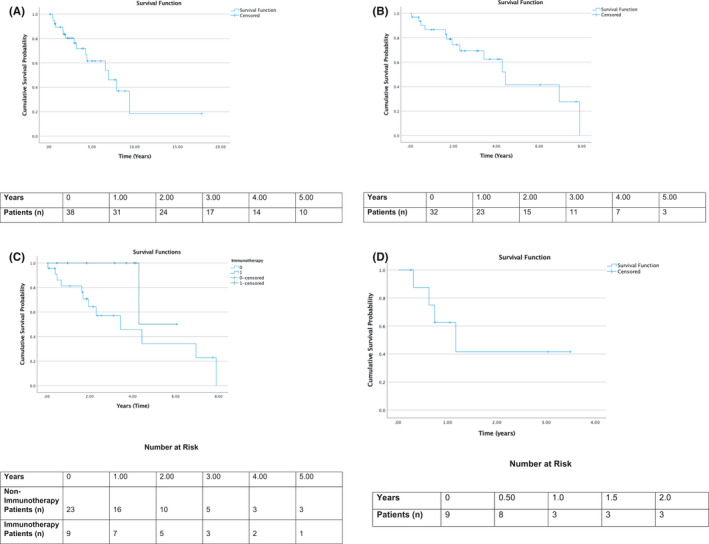
(A) Overall survival of all patients with *CDK12* alteration (*n* = 39). Overall survival defined as the date of diagnosis to death or last follow‐up, whichever came first. Median Survival 6.91 years (95% CI: 3.65–10.22). (B) Overall survival of all patients with *CDK12* alteration who developed metastatic disease at any time (*n* = 32). Overall survival defined as the date of diagnosis of metastatic to death or last follow‐up, whichever came first. Median Survival 4.43 years (95% CI: 3.11–5.74). (C) Overall survival by receipt of checkpoint inhibitor‐containing regimen for all patients with *CDK12* alterations who developed metastatic disease at any time (*n* = 32). Overall survival defined as the date of diagnosis of metastatic to death or last follow‐up, whichever came first. Green line represents patients with metastatic disease having received a checkpoint inhibitor‐containing regimen (*n* = 9). Blue line represents patients with metastatic disease not having received a checkpoint inhibitor‐containing regimen (*n* = 23). (D) Progression‐free survival of checkpoint inhibitor‐containing patients who had metastatic disease (*n* = 9). Progression‐free survival is defined as the time from first‐line immunotherapy start to radiographic progression, clinical progression, death, or last follow‐up. Median progression‐free survival 1.16 (95% CI: 0.32–2.00)

The median follow‐up for the population was 4.01 (95% CI: 1.82–6.21) years from diagnosis to last follow‐up or death. For the total cohort, OS as calculated from the date of diagnosis was 6.94 years (95% CI: 3.65–10.22) (Figure 3A) and 4.43 years (95% CI: 3.11–5.74) from the date of metastases development for those with metastatic disease (*n* = 37) (Figure 3B).

Outcomes for patients treated with checkpoint blockade are delineated in Table 3. The objective response rate to checkpoint inhibition was 60.0% (*n* = 6). At the time of last follow‐up, five patients remained on therapy while five had discontinued treatment due to radiographic progression (*n* = 2), clinical progression (*n* = 1), toxicity (*n* = 1), or other reasons (*n* = 1). The median PFS for patients treated with checkpoint blockade for metastatic disease was 1.16 years (95% CI: 0.32–2.00). We evaluated OS from the date of metastases development in patients having received a checkpoint inhibitor regimen compared to those not having received such therapy. Median OS was not reached for patients treated with checkpoint inhibitor‐containing regimen compared to those not having received such therapy (3.42 years 95% CI: 0.87–5.97) (*p* = 0.089) (Figure 3C).

Of the three patients treated with a PARP inhibitor, no patient achieved an objective response and all developed disease progression with time to progression of 2.0, 2.8, and 3.7 months.

## 4 DISCUSSION

The clinical implications of *CDK12* mutations have been evolving over the last several years, as these genomic aberrations have been detected in a proportion of malignancies. Our study demonstrates that *CDK12*‐altered cancers have a prevalence of 0.88%, which is consistent with previously published reports on *CDK12* genomic alterations. Pan‐cancer studies evaluating the prevalence of *CDK12* alterations have been limited. A similar pan‐cancer analysis, which lacked access to detailed clinical data, demonstrated that the prevalence of *CDK12* genomic alterations was 1.1% across all cancer types, with mutations most frequently seen in prostate, gastrointestinal, and gynecologic malignancies.^7^ These findings are similar to our cohort, with the most commonly mutated tumors being prostate with a frequency of 20% of all *CDK12*‐mutated patients, followed by colorectal cancer. Knowing the prevalence of *CDK12* alterations among various solid tumor types may be helpful for selectively evaluating for these mutations for prognostic and treatment purposes, particularly in the case of prostate cancer where biallelic *CDK12* inactivation is associated with shorter time to biochemical progression and distant metastases.^10^

The ethnic distribution of our cohort was unique in that approximately half of patients with *CDK12*‐altered tumors were non‐White, with Asian/Pacific Islander being the group with the third highest prevalence of *CDK12*‐altered tumors after white individuals. While the prevalence of *CDK12* mutations across ethnic groups has not been fully characterized, there have been some studies highlighting a predisposition for *CDK12*‐mutated breast and ovarian cancers in patients of Eurasian descent.^15^, ^16^ Further studies evaluating the ethnic distribution of genomic alterations are important in bridging disparities gaps in genomic profiling and developing strategies to bring precision medicine to all races and ethnicities.

*CDK12* alterations have also been associated with an increased response to immunotherapy. The mechanism driving *CDK12*‐altered tumor sensitivity to immune checkpoint inhibitors lies in the association between *CDK12*‐loss‐of‐function mutations with a higher focal tandem duplication burden; focal tandem duplications lead to increased production of fusion‐induced neoantigens that can be responsive to PD‐1/PD‐L1 blockade.^10^ This concept was previously demonstrated in the context of prostate and ovarian cancer,^17^ but has more recently expanded to include several different cancer types. In the pan‐cancer analysis by Sokol et al., *CDK12*‐loss‐of‐function was associated with an increased focal tandem duplication burden in all evaluated malignancy subtypes including gastrointestinal, gynecologic, and cancer of unknown primary. While hypothesis generating in nature and not accounting for confounding factors, we observe a potential signal of longer OS in patients with *CDK12*‐mutated cancers having received an immune checkpoint inhibitor‐containing regimen. These data are limited by a relatively small cohort of immunotherapy‐treated patients (10 out of 39) as well as the fact that 4 out of 10 patients who received immunotherapy also had concurrent targeted therapy or chemotherapy, which suggests that the achieved response cannot be attributed to checkpoint blockade alone. With these caveats taken into consideration, the results capture immunotherapy efficacy across a broad spectrum of cancer types including lung, liver, and melanoma.

Interestingly, a subset of patients had co‐occurring *BRCA2* and *CDK12* mutations, raising the question of whether PARP inhibition may be an effective treatment option in this cohort of patients. It has been hypothesized that PARP inhibitors could be effective in *CDK12*‐deficient cancers, as *CDK12* mediates DNA repair via homologous recombination.^5^ In *BRCA*‐mutated triple‐negative breast cancer cells and patient‐derived xenografts, the PARP inhibitor dinaciclib was shown to increase the degree of response for PARP inhibitor‐sensitive models, and reverse homologous recombination and PARP inhibitor resistance.^18^ However, published reports have not shown a pan‐cancer connection between *CDK12* alterations and homologous recombination deficiency (HRD) phenotype. The PROfound trial evaluated the efficacy of olaparib in men with metastatic castrate‐resistant prostate cancer (mCRPC) and a qualifying alteration in genes involved in HRD. The study demonstrated an improvement in PFS with olaparib compared to abiraterone or enzalutamide. However, the proportion of *CDK12*‐altered tumors was 26.2%. In an exploratory analysis in patients with CDK12‐altered tumors, PFS was 5.09 months with olaparib compared to 2.20 months with abiraterone or enzalutamide, though statistical comparison between the groups was not performed.^19^ In our cohort, there was a lack of response to PARP inhibition, which is in concordance with previously published data that also show poor responses to PARP inhibitors in *CDK12*‐altered prostate cancer.^6^ Similarly, HRD has been associated with increased sensitivity to platinum chemotherapy,^20^ and it would be of clinical utility to consider an exploratory analysis of the response of *CDK12*‐altered tumors to platinum compounds.

A strength of our study is that it reflects the real‐world practice of using a multitude of clinical‐grade sequencing assays to identify patients with *CDK12*‐altered tumors. Additionally, our pan‐cancer analysis includes a heterogeneous patient population with a wide spectrum of primary malignancies. However, some limitations are present due to the inherent nature of a retrospective pan‐cancer analysis and our relatively small sample of patients with CDK12‐altered tumors which prevents more robust conclusions. The non‐*CDK12*‐altered dataset had limited granularity in regards to baseline characteristics and clinical outcomes, which prevents comparisons with our *CDK12*‐altered cohort. While variant allele frequency of the *CDK12*‐altered tumors would have been of value to determine its impact on overall tumor biology, this detail was unavailable in the majority of the sequencing assays in our cohort. Evaluating surrogate markers for immunotherapy response, such as MSI/MMRI status, in our *CDK12*‐altered cohort would have been clinically beneficial; however, this was limited by lack of data from earlier genomic reports. The responses to immunotherapy in our cohort could have been influenced by high tumor mutational burden or MSI high status. Further studies evaluating MSI/MMR and *CDK12* alterations are needed to determine whether *CDK12* alterations are independent predictors of immunotherapy response. In addition, only a subset of patients in our cohort were treated with immune checkpoint inhibitors and/or PARP inhibitors, limiting robust comparison of outcomes among patient treated with or without checkpoint blockade.

Given its role in human cancers and regulation of genome stability, *CDK12* is currently being studied as a potential therapeutic target. CDK inhibitors for cancer treatment are on the horizon, with some drugs having multi‐specific CDK inhibitor activity such as dinaciclib,^21^ and others having *CDK12*‐specific inhibition such as THZ531^22^ and SR‐4835.^23^ Dinaciclib has the ability to reverse PARP inhibitor resistance by downregulating homologous recombination DNA repair genes, suggesting that combination therapy with PARP and CDK12 inhibitors may be an effective approach. This combination is currently being studied in a Phase I trial with dinaciclib and veliparib (PARP‐1 inhibitor ABT‐888) for treatment of metastatic solid tumors, and is estimated to complete accrual in December 2021 (available online: http://clinicaltrials.gov, NCT01434316).

In addition, clinical trials evaluating immunotherapy response in *CDK12*‐mutated cancers are underway. The IMPACT trial is an ongoing study investigating whether *CDK12*‐mutated mCRPC is more susceptible to nivolumab and ipilimumab (NCT03570619). There is also the Phase II study of abemaciclib and atezolizumab in mCRPC (NCT04751929), as well as durvalumab and olaparib in prostate cancer patients with high neoantigen load (NCT 04336943). Overall, *CDK12* is a promising targetable biomarker that may be predictive of immune checkpoint inhibitor sensitivity, and also play a role in clinical decision making for selective genomic sequencing and its therapeutic implications. The prevalence of *CDK12* alterations is rare and conclusions cannot be drawn based on our current data as to whether pan‐screening for *CDK12* is of clinical utility. Impact trial testing of immunotherapy in patients with *CDK12*‐mutated malignancies will help better guide decision making around *CDK12* testing.

## 5 CONCLUSION

This study is one of the few pan‐cancer analyses of *CDK12* alterations demonstrating that *CDK12* alterations are rare events across different cancer types. *CDK12* mutations were associated with responses to immunotherapy, suggesting that *CDK12* may be a predictive biomarker of immune checkpoint inhibitor response in addition to being a marker with targetable therapeutic potential.

## CONFLICT OF INTEREST

The authors declare that they have no competing interests.

## AUTHORS’ CONTRIBUTION

All authors contributed to the study design and research of this article. SL provided details for molecular genetic studies on our patient cohort. EP, AC, JJ‐D, DP, and RM participated in the statistical analysis and drafted the manuscript. All authors read and approved the final manuscript.

## ETHICAL APPROVAL

Ethical approval was obtained from the Institutional Review Board at UCSD prior to commencing this study.

## Supporting information

Table S1‐S2

## Data Availability

The data that supports the findings of this study are available in the supplementary material of this article.
