# Supplementary material for: Analysis of CDK12 alterations in a pan‐cancer database
Source: Cancer Med. 2021 Dec 12;11(3):753–63. doi: 10.1002/cam4.4483 (PMC8817093; doi:10.1002/cam4.4483)
Supplement: Supplementary file 1 — Table S1‐S2 [file CAM4-11-753-s001.docx]

**Supplemental Material**

**Supplement Table 1.** Tumor characteristics of patients without *CDK12* alterations (n=4,947).

| **Histology** | **Number** | **Percent** |
| --- | --- | --- |
| Adenocarcinoma | 2986 | 60.4% |
| Squamous Cell Carcinoma | 460 | 9.3% |
| Urothelial Carcinoma | 76 | 1.5% |
| Neuroendocrine Carcinoma | 119 | 2.4% |
| Small Cell Carcinoma | 35 | 0.7% |
| Melanoma | 233 | 4.7% |
| Lymphoid Malignancy | 469 | 9.5% |
| GIST | 110 | 2.2% |
| Sarcoma | 162 | 3.3% |
| Other | 297 | 6.0% |
|  |  |  |
| **Primary Cancer Site** |  |  |
| Lung | 581 | 11.7% |
| Colorectal | 511 | 10.3% |
| Breast | 440 | 8.9% |
| Hepatobiliary | 495 | 10.0% |
| CNS | 241 | 4.9% |
| Prostate | 201 | 4.1% |
| Melanoma | 196 | 4.0% |
| MDS/Leukemia | 332 | 6.7% |
| Lymphoma | 169 | 3.4% |
| Gastroesophageal | 271 | 5.5% |
| Ovarian | 160 | 3.2% |
| Small Intestine | 248 | 5.0% |
| Head and neck | 131 | 2.6% |
| Sarcoma | 129 | 2.6% |
| Non-melanoma skin | 127 | 2.6% |
| Uterine | 109 | 2.2% |
| Thyroid | 94 | 1.9% |
| Bladder | 90 | 1.8% |
| Kidney | 89 | 1.8% |
| Salivary Gland | 38 | 0.8% |
| Cervix | 34 | 0.7% |
| Multiple myeloma | 29 | 0.6% |
| Anal | 29 | 0.6% |
| Other | 203 | 4.1% |
|  |  |  |
| **Disease Status at Last Follow-Up** |  |  |
| Metastatic | 2999 | 60.6% |
| Non-Metastatic | 1937 | 39.2% |
| Unknown | 11 | 0.2% |
|  |  |  |
| **Test Type*** |  |  |
| cfDNA |  |  |
| Guardant 360 | 648 | 13% |
| FoundationOne Heme | 742 | 15% |
| FoundationOne Liquid | 250 | 5% |
| Tissue |  |  |
| Tempus xE | 29 | 0.6% |
| Tempus xO | 81 | 2% |
| Tempus xT | 778 | 16% |
| FoundationOne | 2771 | 56% |
| FoundationOne CDx | 639 | 13% |

GIST=Gastrointestinal stromal tumor; CNS=Central nervous system; MDS=Myelodysplastic syndrome.

*Number of tests reflects the total assays that were run and includes individual patients who may have had multiple tests for blood and tissue analyses

**Supplement Table 2.** Listing of unique *CDK12* alterations identified in each patient.

| **Test** | **Mutation** | **Type** |
| --- | --- | --- |
| Foundation One | *CDK12*(NM_015083) duplication intron 8 - exon 13 | Duplication |
| Foundation One | *CDK12* C1009fs*3 | Frameshift |
| Foundation One | *CDK12* T541fs*66 | Frameshift |
| Foundation One | *CDK12* T1463fs*30+ | Frameshift |
| Foundation One | *CDK12* S169fs*31 | Frameshift |
| Foundation One | *CDK12* F336fs*1 | Frameshift |
| Foundation One | *CDK12* Q244s*93 | Frameshift |
| Foundation One | *CDK12* T1463fs*30+ | Frameshift |
| Foundation One | *CDK12* R329fs*21 | Frameshift |
| Foundation One | *CDK12* T206fs*125 | Frameshift |
| Foundation One | *CDK12* L760fs*2 | Frameshift |
| Guardant | *CDK12* R981fs | Frameshift |
| Guardant | *CDK12* D416fs | Frameshift |
| Guardant | *CDK12* P577fs | Frameshift |
| Tempus | *CDK12* p.S200fs Frameshift | Frameshift |
| Foundation One | *CDK12* S323_P324>*SP | Missense |
| Tempus | *CDK12* p.E751* Splice region variant - LOF | Missense |
| Foundation One | *CDK12* R979* | Non-Sense |
| Foundation One | *CDK12* Q1368* | Non-Sense |
| Foundation One | *CDK12* R979* | Non-Sense |
| Foundation One | *CDK12*E205* | Non-Sense |
| Foundation One | *CDK12* Q598* | Non-Sense |
| Foundation One | *CDK12* Y279* | Non-Sense |
| Foundation One | *CDK12* R1051* | Non-Sense |
| Foundation One | *CDK12* R983* | Non-Sense |
| Guardant | *CDK12* G239* Non-sense | Non-Sense |
| Tempus | p.Q944* Stop gain - LOF | Non-Sense |
| Tempus | p.Y327*Stop gain - LOF | Non-Sense |
| Foundation One | *CDK12* rearrangement | Rearrangement |
| Foundation One | *CDK12* splice site 2666+1G>A | Splice |
| Foundation One | *CDK12* splice site 2420-1G>A | Splice |
| Foundation One | *CDK12* splice site 2610-20_2610-1>T | Splice |
| Foundation One | *CDK12* Copy Gain | Splice |
| Foundation One | *CDK12* rearrangement intron 11 | Translocation |
| Foundation One | *CDK12* (NM_015083) rearrangement intron 4 | Truncation |
| Foundation One | *CDK12* truncation | Truncation |
| Foundation One | *CDK12* truncation intron 6 | Truncation |
| Foundation One | *CDK12* rearrangement intron 7 | Truncation |
| Foundation One | *CDK12* truncation exon 8 | Truncation |
| Foundation One | *CDK12*-LARP4B rearrangement | Truncation |
